# The Prevalence of Celiac Disease in a Fracture Liaison Service Population

**DOI:** 10.1007/s00223-020-00725-z

**Published:** 2020-07-28

**Authors:** Irma J. A. de Bruin, Lisanne Vranken, Caroline E. Wyers, Robert Y. van der Velde, Thera A. M. Trienekens, Sjoerd Kaarsemaker, Heinrich M. J. Janzing, Frank L. Wolters, Siep Wouda, Piet P. M. M. Geusens, Joop P. W. van den Bergh

**Affiliations:** 1 Department of Internal Medicine, VieCuri Medical Center, Tegelseweg 210, PO Box 1926, 5900 BX Venlo, The Netherlands; 2 NUTRIM School of Nutrition and Translational Research in Metabolism, Department of Internal Medicine, Maastricht University Medical Centre+ (MUMC+), PO Box 616, 6200 MD Maastricht, The Netherlands; 3 Department of Medical Microbiology, VieCuri Medical Center, Venlo, The Netherlands; 4 Department of Orthopaedic Surgery, VieCuri Medical Center, Venlo, The Netherlands; 5 Department of Surgery, VieCuri Medical Center, Venlo, The Netherlands; 6 Department of Gastro-Enterology, VieCuri Medical Center, Venlo, The Netherlands; 7 Department of Pathology, VieCuri Medical Center, Venlo, The Netherlands; 8 Department of Internal Medicine, Subdivision Rheumatology, CAPHRI, Maastricht University Medical Centre+ (MUMC+), PO Box 616, 6200 MD Maastricht, The Netherlands; 9 Biomedical Research Centre, Hasselt University, Agoralaan–gebouw D, 3590 Diepenbeek, Belgium

**Keywords:** Fracture liaison service, Celiac disease, Osteoporosis, Fractures

## Abstract

Celiac disease (CD) is a known risk factor for osteoporosis and fractures. The prevalence of CD in patients with a recent fracture is unknown. We therefore systematically screened patients at a fracture liaison service (FLS) to study the prevalence of CD. Patients with a recent fracture aged ≥ 50 years were invited to VieCuri Medical Center’s FLS. In FLS attendees, bone mineral density (BMD) and laboratory evaluation for metabolic bone disorders and serological screening for CD was systematically evaluated. If serologic testing for CD was positive, duodenal biopsies were performed to confirm the diagnosis CD. Data were collected in 1042 consecutive FLS attendees. Median age was 66 years (Interquartile range (IQR) 15), 27.6% had a major and 6.9% a hip fracture, 26.4% had osteoporosis and 50.8% osteopenia. Prevalent vertebral fractures were found in 29.1%. CD was already diagnosed in two patients (0.19%), one still had a positive serology. Three other patients (0.29%) had a positive serology for CD (one with gastro-intestinal complaints). In two of them, CD was confirmed by duodenal histology (0.19%) and one refused further evaluation. The prevalence of biopsy-proven CD was therefore 0.38% (4/1042) of which 0.19% (2/1042) was newly diagnosed. The prevalence of CD in patients with a recent fracture at the FLS was 0.38% and within the range of reported prevalences in the Western-European population (0.33–1.5%). Newly diagnosed CD was only found in 0.19%. Therefore, standard screening for CD in FLS patients is not recommended.

## Introduction

Celiac disease (CD) is an autoimmune enteropathy induced by dietary proteins in wheat, rye, and barley. The presentation of symptoms widely varies. In 2012, the following Oslo definitions for CD were stated: ‘classical CD presents with signs and symptoms of malabsorption. Besides malabsorption, other symptoms of diarrhea, steatorrhea, weight loss, or growth failure are required. Non-classical CD presents with gastro-intestinal symptoms and extra intestinal manifestations, but without signs and symptoms of malabsorption and diarrhea. Subclinical CD is disease below the threshold of clinical detection without signs or symptoms sufficient to trigger CD testing in routine practice’ [1]. It was demonstrated that in a period of 15 years (1998–2012) an increasing part of the CD patients had a subclinical CD or a non-classical phenotype instead of the classical CD phenotype [2].

CD is a known risk factor for osteoporosis and fractures, with a RR of 1.3–1.9 for fractures [3–6]. Malabsorption of calcium and vitamin D deficiency leads to secondary hyperparathyroidism. General malnutrition and underweight also result in a reduced bone mineral density (BMD) [3, 7]. Further, hypogonadism associated with CD might also affect bone metabolism [7, 8]. Chronic inflammation and release of proinflammatory cytokines lead to an increase in osteoclastic bone resorption [9]. Appropriate treatment of CD relieves symptoms and can improve BMD [10–12]. However, after diagnosis of CD the increased risk of fractures persists [6]. It was demonstrated that the increased fracture risk remained 20 years after diagnosis of CD [13].

The worldwide prevalence of CD based on serologic tests is reported to be 1.4% and of biopsy-proven CD 0.7% [14]. In the general unselected Northern American and Western-European populations, the prevalence of CD is close to 1% and in Northern European countries it is slightly higher, around 1–1.5% [15]. Rostami et al*.* reported a prevalence of biopsy-proven CD of 3 per 1000 persons in a Dutch population of healthy blood donors [16]. In high-risk populations, such as in type 1 diabetes patients and first-degree relatives of patients with CD, the prevalence of CD is estimated to be higher than in the general population: 3–6% in patients with type 1 diabetes and up to 20% in first-degree relatives of CD patients [15]. Based on a clinical review the prevalence of CD was estimated between 2 and 3% in low-BMD populations [17]. A recent meta-analysis showed a prevalence of biopsy-proven CD of 1.6% in patients with osteoporosis [18].

From 1999 onwards, fracture liaison services (FLSs) were initiated aiming at reduction of subsequent fracture risk in high-risk patients, namely those who sustained a recent fracture [19, 20]. Besides screening for osteoporosis, screening for metabolic bone disorders is recommended in FLS patients [21–24]. In general, laboratory evaluation of secondary causes of osteoporosis and metabolic bone disorders does not include screening for CD. Rios et al*.* concluded that there is no evidence for routine screening for CD in all patients with low BMD [17].

To our knowledge, the prevalence of CD in an FLS population has not been studied so far. This might be important, given the fact that CD is associated with increased fracture risk [6, 13]. Therefore, our aim was to study the prevalence of CD in an FLS population. In view of the increased risk of fractures in CD, we hypothesized that CD would be more frequent in the FLS population than the reported prevalence in the total population.

## Methods

### Fracture Liaison Service

We conducted a retrospective cohort study in patients with a recent clinical fracture, aged 50–90 years, visiting the FLS of a regional teaching hospital for fracture risk evaluation (VieCuri Medical Centre, Venlo, The Netherlands). Patients with a skull fracture, patients older than 90 years and patients with an active malignancy were excluded.

FLS attendees received a detailed questionnaire for evaluation of clinical risk factors for fractures, medical history, medication, previous fractures, and calcium intake and were scheduled for dual X-ray absorptiometry (DXA) measurement and a blood test. A visit at the outpatient clinic was scheduled after completion of these tests. At this visit height and weight were assessed, the questionnaire was evaluated and additional questions were asked. If laboratory results were abnormal, additional investigations were performed for detailed evaluation of newly diagnosed disorders when necessary. Depending on the BMD results, calcium intake and serum 25-hydroxyvitamin D [25(OH)D] levels, patients were treated with calcium and vitamin D supplements, and anti-osteoporosis medication according to the Dutch guidelines for treatment of osteoporosis [25].

Index fractures were classified according to the Center classification: hip, major (vertebra, pelvis, distal femur, proximal tibia, multiple rib, and proximal humerus), minor (all others except major and finger & toe fractures), and finger & toe fractures [26].

### DXA and VFA

BMD in the left or right hip and the lumbar spine was determined using dual-energy X-ray absorptiometry (DXA) with the Hologic QDR 4500 (Hologic, Bedford, MA, USA). Diagnosis of osteoporosis was based on the World Health Organization criteria for BMD [27], as provided by the manufacturer for women and men and which are based on the National Health and Nutrition Examination Survey III database. T-score calculations were done for women with a female and for men with a male reference population, as provided by the manufacturer. Patients were classified according to the lowest value of T-score in total hip, femoral neck, or lumbar spine: osteoporosis as a T-score of −2.5 or less, osteopenia as a T-score between −2.5 and −1.0, and normal BMD as a T-score of −1.0 or higher.

Assessment of vertebral fractures was performed via vertebral fracture assessment (VFA). Vertebral fractures were graded according to the grading of Genant et al*.* as grade 1, 20–24% reduction in vertebral body height at the anterior, mid, or posterior location; grade 2, 25–39%; or grade 3, ≥ 40% reduction, respectively [28].

### Screening and Diagnosis of Celiac Disease

In accordance with the Dutch and American guidelines of CD, as first-line test the serological screening for CD was performed in this low-risk cohort [29, 30]. Serological screening consisted of measurement of serum IgA and IgA tissue transglutaminase antibodies (tTG). Serum IgA tTG values were measured using the ELiA Celikey IgA kit (Phadia AB, Uppsala, Sweden). The sensitivity and specificity for this test are 96% and 99%, respectively. Anti-tTG IgA can only be assessed accurately if an IgA deficiency is excluded. Since IgA deficiency is more prevalent in patients with CD than in the general population, IgA was evaluated in all patients in addition to anti-tTG IgA [31]. If the IgA titer was less than 0.2 g/l, IgG tTG antibodies were measured. An anti-tTG IgA titer of 8 U/ml or more was considered as a positive test result suspicious for CD. In patients with a positive anti-tTG IgA test, without a history of positive CD serology, an additional anti-endomysial IgA (EMA) (SciMedX IFA, Libra Diagnostica) test was performed as confirmation test. The sensitivity and specificity for this test vary between 95–99% and 97–98%, respectively.

In the case of positive anti-tTG and anti-EMA test result, patients were referred to the gastroenterologist for a duodenoscopy with duodenal biopsies. Histopathological examination was performed according to the Modified Marsh criteria [32, 33]. This classification describes the histopathology of CD, based on three aspects: microscopic enteritis (increased intraepithelial lymphocyte (IEL) count), crypt hyperplasia and villus atrophy.

CD was diagnosed in case of a positive anti-tTG IgA and anti-endomysial IgA serology in combination with a duodenal biopsy with characteristics of CD that conform to the Modified Marsh classification.

Since the positive serology for CD can normalize with a gluten-free diet, CD cannot be excluded with negative serology. In addition to the serology, we verified the past medical history. In the case of a positive medical history for celiac disease, the medical record of the patient was checked for positive serology and duodenal biopsies in the past.

### Statistics

Data were analyzed using descriptive statistics (mean, median) by IBM SPSS statistics 24.

This retrospective cohort study was approved by the medical research ethics committee of the Academic Hospital Maastricht/University Maastricht (METC 2020-1508).

## Results

From a total of 2376 consecutive patients with a recent fracture who were invited at the FLS, 1042 patients (43.9%) actually attended the FLS. All FLS attendees were screened for CD. As shown in Table 1, median age of the study population was 66.0 years (Interquartile range (IQR) 15) and 719 (69.0%) were women. The majority (54.4%) had a minor fracture, 27.6% a major fracture, 6.9% a hip fracture, and 11% a finger or toe fracture. Osteoporosis was diagnosed in 26.4%, osteopenia in 50.8%, and 22.8% had a normal BMD. VFA analysis showed at least one prevalent vertebral fracture (at least one grade 1, 2, or 3) in 303 patients (29.1%) and at least one grade 2 or 3 vertebral fracture in 190 patients (18.2%). Vitamin D deficiency (serum 25(OH)D < 50 nmol/l) was present in 40%. The median serum calcium (corrected for serum albumin) was 2.42 mmol/l (IQR 0.10). None of the patients had hypocalcemia (serum calcium corrected for albumin < 2.10 mmol/l) and 69 patients had hypercalcemia with a corrected serum calcium of > 2.55 mmol/l. The median parathyroid hormone (PTH) was 5.3 pmol/l (IQR 3.5) (reference range: 2.2–10.0 pmol/l). The median hemoglobin was 8.5 mmol/l (IQR 0.9). In total, 75 patients had anemia, of which 35 men with a hemoglobin of < 8.0 mmol/l, and 40 women with a hemoglobin of < 7.2 mol/l. The self-reported calcium intake was 780.0 mg per day (IQR 387.0).

**Table 1:** Baseline characteristics FLS population (*n* = 1042)

Age (years)	66.0 (IQR 15)
Gender (% females)	69.0%
Length (cm)^a^	165.5 (IQR 12)
Weight (kg)^a^	75.0 (IQR 19.7)
BMI (kg/m^2^)^a^	26.8 (IQR 6.2)
Fracture (center) (%)	
Finger and toe	11.0%
Minor	54.4%
Major	27.6%
Hip	6.9%
BMD^b^ (%)	
Normal BMD	22.8%
Osteopenia	50.8%
Osteoporosis	26.4%
Vertebral fractures (VFA)^c^ (%)	
At least one grade 1, 2, or 3 (%)	29.1%
At least one grade 2 or 3 (%)	18.2%
Grade 1	15.1%
Grade 2	13.8%
Grade 3	6.5%
Self-reported calcium intake mg/day^d^	780.0 (IQR 387.0)
Calcium (mmol/l)	2.42 (IQR 0.10)
Phosphate (mmol/l)	1.13 (IQR 0.22)
Albumin (g/l)	40 (IQR 4)
PTH (pmol/l)	5.3 (IQR 3.5)
Hemoglobin (mmol/l)	8.5 (IQR 0.9)
IgA < 0.2 g/l (%)	0.19%
Anti-tTG IgA > 8 U/ml (%)	0.38%
Vitamin D < 50 nmol/l	40%

*BMI* body mass index, *BMD* bone mineral density, *VFA* vertebral fracture assessment, *PTH* parathyroid hormone, *anti*-*tTG* anti-tissue transglutaminase

^a^Data missing of 6 patients

^b^Data missing of 2 patients

^c^Data missing of 3 patients

^d^Data missing of 22 patients

### Serologic Testing and Histopathological Testing for CD

Two out of 1042 patients had IgA deficiency (0.19%) and were further tested with anti-tTg IgG which was negative in both. Anti-tTG IgA serology was positive in 4 (0.38%) patients (patient A–D, Table 2). In one patient with previously positive CD serology and biopsy-proven CD, current anti-tTG IgA serology was negative (patient E, Table 2). Of the four patients with positive anti-tTG IgA serology, one patient was already diagnosed with biopsy-proven CD (patient D). The tTG titers varied between 1.6 and 91.4 U/ml (normal range < 8.0 U/ml). The three new patients with positive serology all had a positive IgA anti-EMA.

**Table 2:** Characteristics of the FLS patients with positive tTG serology or known celiac disease

	Patient A	Patient B	Patient C	Patient D	Patient E
Age	64	50	68	76	60
Gender	Female	Female	Female	Male	Male
Length (cm)	163.3	162	164.6	178	183.5
Weight (kg)	81.9	62.2	69.7	85	86
BMI	30.71	23.7	25.73	26.83	25.54

Fracture (center)	Minor	Minor	Major	Minor	Minor
Fracture location	Radial head	Distal radius	Tibial plateau	Tarsal bone	Midshaft ulnar
BMD (T-scores)					
Lumbar spine	− 2.6	− 2.9	− 0.2	− 1.7	− 0.7
Femoral neck	− 2.4	− 2.2	− 1.2	− 2.3	− 0.2
Total hip	− 2.2	− 0.8	− 0.4	− 1.8	0

BMD	Osteoporosis	Osteoporosis	Osteopenia	Osteopenia	Normal BMD
Vertebral fractures (VFA)	No VF	No VF	Th11 grade 1	Th12 grade 3	No VF
Self-reported calcium intake mg/day	711	369	1169	1028	1020
Calcium (mmol/l)	2.35	2.46	2.43	2.42	2.34
Phosphate (mmol/l)	1.30	1.25	1.17	1.03	1.00
Albumin (g/l)	39	41	38	34.0	41.0
25-OH vitamin D (nmol/l)	75	101	29	83	60
PTH (pmol/l)	4.4	4.0	7.8	18.0	5.0
Hemoglobin (mmol/l)	7.4	8.0	8.3	8.2	8.8
IgA (g/l)	6.38	2.72	2.14	4.03	4.43
IgA anti-tTG (U/ml)	91.4	36.5	24.6	37.4	1.6
IgA anti-EMA	Positive	Positive	Positive	Not performed	Not performed
Duodenal biopsy	–	Marsh 3c	Marsh 3a	Marsh 3b	Marsh 3b
	Uncertain celiac disease	Proven celiac disease (new)	Proven celiac disease (new)	Proven celiac disease (known)	Proven celiac disease (known)
Osteoporosis treatment	Refused treatment	Start alendronic acid	No indication for treatment	Treated with risedronic acid	No indication for treatment

*FLS* fracture liaison service, *BMI* body mass index, *BMD* bone mineral density, *VFA* vertebral fracture assessment, *PTH* parathyroid hormone, *IgA* Immunoglobulin A, *IgA* anti-tTg anti-tissue transglutaminase antibodies, *IgA* anti-EMA anti-endomysial antibodies

Based on the positive serology with an anti-tTg IgA of 91.4 U/ml and positive anti-EMA IgA, patient A was suspected for having CD, but she refused further examination and treatment. The two other new patients with positive serology were further evaluated by the gastroenterologist. Duodenal biopsies confirmed the diagnosis of CD in both patients (patients B and C) with biopsy results of Marsh 3c and 3a histology, respectively. Patients D and E with known CD had both previous biopsy results with Marsh 3b histology. The prevalence of biopsy-proven CD in our FLS cohort was therefore 0.38% (4/1042), with newly diagnosed, biopsy-proven CD in 0.19% (2/1042).

### Symptoms and Signs in CD Patients at the FLS

At the outpatient clinic, only one patient (B) had gastro-intestinal complaints, namely loose stools. In two patients, CD was diagnosed 3 and 9 years before the visit at the FLS, based on iron deficiency anemia in patient D and gastro-intestinal complaints in patient E. One patient (C) had a low vitamin D, the others had a normal vitamin D level (reference range: 50–140 nmol/l). In patient D, the PTH was 18 pmol/l with normal calcium and vitamin D levels, which points at a secondary hyperparathyroidism possibly due to malabsorption. BMD was normal in one patient (Patient E), two patients had osteopenia (C and D), and two had osteoporosis (A and B). Prevalent VFs were found in two patients (one grade 1 vertebral fracture in patient C and one grade 3 vertebral fracture in patient D).

### Treatment of CD and Osteoporosis

Patient A refused treatment for CD and osteoporosis. Patients B and C started a gluten-free diet and patients D and E already had a gluten-free diet. All four patients (B–E) had regular visits at the outpatient clinic of the gastroenterologist. Treatment with oral bisphosphonates was started in patient B because of the diagnosis of osteoporosis after a recent major osteoporotic fracture at the distal radius, and patient D already received treatment with risedronic acid. Patients C and E did not receive anti-osteoporosis treatment according to the Dutch guidelines (indication for treatment: T-score < = − 2.5 and/or a moderate or severe vertebral fracture).

## Discussion

In this cohort of 1042 consecutive FLS patients, four patients had biopsy-proven CD. In two patients, CD was already known (0.19%) and in two patients CD was newly detected (0.19%) by systematic serologic testing. One patient was suspected of having CD but refused further analysis. Since we based the diagnosis of CD on well-established criteria of positive CD serology and abnormal duodenal histology [34, 35], the diagnosis of CD could not be confirmed in the fifth patient.

The prevalence of CD in general unselected Western populations is close to 1% and in the general unselected Northern European populations it is approximately 1–1.5% [15]. In a Dutch population of healthy blood donors, the reported prevalence of biopsy-proven CD was 0.33% [16]. The prevalence of 0.38% in our Dutch FLS cohort was somewhat lower than reported in the general Western population and in the same range as in the Dutch healthy blood donors, but it was lower than most of the reported prevalences of CD in osteoporosis patients [15, 16]. Studies of the prevalence of CD in populations with osteoporosis showed varying prevalences. Legroux-Gérot et al*.* did not demonstrate positive CD serology (anti-tTG) in a cohort of 140 patients with osteoporosis [36]. Nuti et al*.* found a positive CD serology in 24 (9.4%) patients with osteoporosis, but only in 10 patients a biopsy was done to prove CD [37]. Gonzalez et al. reported a prevalence of biopsy-proven CD of 0.8% in osteoporosis patients which was demonstrated to be equal to the prevalence in the healthy population [38]. A recent meta-analysis reported a prevalence of biopsy-proven CD of 1.6% among 3188 individuals with osteoporosis [18]. These results of varying prevalences might be explained by the different populations and importantly also by the different screening tests which were used for the diagnosis of CD. Hill et al*.* described differences in sensitivity and specificity of the serological tests [39].

The prevalence of CD in patients with a recent fracture at the FLS has not been studied before. This might be important, given the fact that CD is associated with increased fracture risk [6, 13]. Hjelle et al*.* studied the prevalence of CD in 400 patients aged 40 years or older with a distal radius or ankle fracture compared to community-based controls [40]. The diagnosis of CD was based on serological screening of anti-tTg IgA in combination with histology from duodenal biopsy or a previous diagnosis of CD. Three patients with a fracture had known CD and among all patients with a fracture, 10 had positive serological screening, and nine of them underwent duodenal biopsies. Six patients with a fracture were newly diagnosed with biopsy-proven CD (a prevalence of 1.5%) and in total nine patients had CD, a prevalence of 2.25%. In the control group of 197 patients, four had biopsy-proven CD (2.0%) and in one patient with positive serology no biopsy was performed. Serology was only positive in two controls because of the use of a gluten-free diet in three known CD patients [40]. In this study, in patients with a fracture a positive anti-tTg IgA was more prevalent than in controls, but the prevalence of biopsy-proven CD in the fracture cohort was comparable to the control cohort. Compared to our study, the prevalence of CD was higher, although it was only studied in patients with a distal radius and ankle fracture. In addition, the prevalence in the control group was higher than in the general Western population. The prevalence of CD in our Dutch FLS cohort was comparable to the reported prevalence of CD in healthy Dutch blood donors. Hence, based on the study of Hjelle et al*.* and our findings, the prevalence of CD is not higher compared to healthy subjects without fractures. Therefore, we do not recommend standard screening for CD in all patients with a recent fracture.

The two patients with known CD of our cohort had signs or symptoms of CD, namely iron deficiency anemia and gastro-intestinal complaints at the time of the CD diagnosis years ago. The three patients with new positive serology were not clinically suspected and therefore did not present as the classical phenotype, but seemed to have a subclinical or non-classical CD. It has been demonstrated that in the past years an increasing part of the CD patients has a subclinical CD or a non-classical phenotype instead of the classical CD phenotype [2].

Larussa et al*.* reported that a low BMD was found in 38–72% of patients at time of diagnosis of CD and in 9–47% of patients on a gluten-free diet [41]. In small a cohort patients aged > 65 years with a new diagnosis of CD, osteoporosis was found in 67% of men and 70% of the women [42]. Appropriate treatment of CD relieved symptoms and can improve BMD [10–12]. Improvement of BMD with a gluten-free diet could also be achieved in patients aged > 65 years [42].

In our cohort, of the five with positive serology or biopsy-proven CD, two had osteoporosis (40%), two had osteopenia (40%), and one had a normal BMD. One patient had a major fracture at the tibia (patient C). On the other hand, only 0.73% (2/275) of all patients with osteoporosis and only 0.35% (1/288) of all patients with a major fracture and 0.66% (2/303) of all patients with a prevalent vertebral fracture had CD. Therefore, fracture or BMD characteristics cannot be used to distinguish patients with possible CD from patients without CD.

The cost of serologic CD screening (IgA and anti-tTG IgA) at our hospital was €22,42 per patient and for the confirmation test (anti-endomysial IgA) €32,41. Given the low prevalence of CD in our FLS cohort, the number needed to screen in order to diagnose one patient with CD is 261. In FLS patients with osteoporosis, the number needed to screen was 138 and in patients with a major osteoporotic fracture or a prevalent VF it was 288 and 152, respectively. Based on these findings, we believe screening for CD in FLS patients is not recommended, which is in line with Laszkowska et al*.* who also reported that routinely screening for CD in osteoporosis is not recommended because of the low prevalence of CD [18]. Nevertheless, it will be still indicated to analyze the presence of CD in FLS patients with laboratory results, comorbidity or symptoms suggestive of CD. This is in line with the recommendation of Rios et al*.* of a targeted case finding approach [17]. Further, in younger patients with osteoporosis (aged < 50 years) it is indicated to perform serological screening for CD because underlying causes of osteoporosis or metabolic bone diseases are more prevalent in these patients [43].

This study has several limitations. Approximately 50% of invited patients with a recent fracture actually attended the FLS. It is therefore unknown whether the prevalence of CD in the attenders is comparable of those of the non-attenders. There might be a selection bias since patients with known CD will have standard DXA evaluations in the Netherlands according to the guidelines [29], which could have led to a higher proportion of CD patients in FLS non-attenders. Furthermore, one patient with positive serology refused further analysis for CD. Therefore, it was not possible to confirm the positive serology with biopsies to diagnose CD properly. Thirdly, we did not check if patients were eating a gluten-free diet. The use of a gluten-free diet can normalize serology for CD. Over the past years, there is an increase in people consuming a gluten-free diet. This increase can be explained partially because of people without CD avoiding gluten, for example as ‘healthy’ lifestyle [44, 45]. Fourthly, at our FLS there was no systematic evaluation of gastro-intestinal symptoms and no standard evaluation of the family history of CD. Therefore, we could not calculate the number needed to screen in FLS patients with gastro-intestinal complaints, nor could we calculate the number needed to screen in the high-risk patients with a first-degree relative with CD. One of the strengths of this study was that serological screening consisted of measurement of serum IgA and IgA tissue transglutaminase antibodies (tTG) with a sensitivity and specificity for this test of 96% and 99%, respectively. Further, this was the first study for CD screening in a general FLS population.

## Conclusion

The prevalence of CD in patients with a recent fracture at the FLS was 0.38% and within the range of reported prevalences in the Western-European population (0.33–1.5%). Newly diagnosed CD was only found in 0.19%. We therefore believe that standard screening for CD in FLS patients is not recommended.

## Data Availability

Not applicable.
